# Microglia regulate the health of central nervous system through myelin

**DOI:** 10.1186/s43556-023-00124-4

**Published:** 2023-05-15

**Authors:** Xin Niu, Fangfang Zhou, Long Zhang

**Affiliations:** 1 MOE Laboratory of Biosystems Homeostasis & Protection and Innovation Center for Cell Signaling Network, Life Sciences Institute, Zhejiang University, 310058 Hangzhou, PR China; 2 Institutes of Biology and Medical Science, Soochow University, 215123 Suzhou, PR China; 3 International Biomed-X Research Center, School of Medicine, Second Affiliated Hospital of Zhejiang University, Zhejiang University, 310058 Hangzhou, PR China

Myelin is essential for the health of the central nervous system, while basic mechanisms coordinating the formation, growth, and integrity of myelin in the central nervous system remain unknown. Although macrophages are associated with myelin health, the macrophage populations regulating myelin formation and the function of them in myelin health needs to be further explored [1]. In a recent study, McNamara et al. found that the unique lineage of macrophages, microglial cells, regulate the growth and integrity of myelin.

Learning and memory are inseparable from the function of oligodendrocytes, which generated later in development. However, long-term consolidation of related information involves an increase in myelin, and neuronal axons covered with myelin which have good structural integrity [2, 3]. With aging and onset of neurodegenerative diseases, hypermyelination occurs, and the uncompressed area expands, thickens, and eventually denatures, resulting in the loss of myelin integrity [4]. Therefore, there is an urgent need to explore the key signaling pathways that regulate myelin formation, growth, and integrity to provide possible therapeutic targets for myelin disorders such as aging, and neurodegenerative diseases including dementia, Parkinson’s disease and Alzheimer’s disease.

To investigate the specific role of microglia in central nervous system health, the authors used a *Fire*^*Δ/Δ*^ mouse model with microglia-specific deletion while other central nervous system macrophages were unaffected (Fig. 1). The authors found that there was no significant difference in the number of OLIG2^+^ oligodendrocyte lineages or the number of myelin axons, regardless of axon diameter. However, 1-month-old *Fire*^*Δ/Δ*^ mice exhibited abnormal myelin structure indicative of hypermyelination. Small-diameter axons showed enlarged areas of the inner tongue, which located in the innermost and uncompact part of the myelin sheath. Large-diameter axons showed increased myelin thickness. Moreover, axon spheroids that cause impaired axonal transport were observed. As the phenotypes of these myelin structures described above often lead to cognitive deficits, the authors further examined changes in cognitive function in mice. Memory task experiments showed that *Fire*^*Δ/Δ*^ mice had impaired cognitive flexibility, and the number of myelin axons was significantly reduced after the completion of the cognitive task. These results suggest that microglia are necessary for myelin growth, which in turn affects the related cognitive functions. In 6-month-old *Fire*^*Δ/Δ*^ mice, the authors found that loss of microglia resulted in reduced inner tongue size, thinner myelin, and increased areas of substantial demyelination and patchy demyelination. These results further prove that the absence of microglia can promotes demyelination in the central nervous system. To further verify the role of microglia in myelin maintenance, the authors added the CSF1R inhibitor PLX5622 to the diet of 2-month-old *Fire*^*+/+*^ mice. PLX5622 treatment resulted in an enlarged inner tongue and thicker myelin followed by patchy demyelination, suggesting that microglia are essential for myelin maintenance after myelination. Moreover, the authors analyzed samples from patients with adult-onset leukoencephalopathy with axonal spheroids and pigmented glia (ALSP). ALSP is characterized by axon and glial damage and is a rare degenerative disease of white matter. Autosomal dominantly inherited mutations in CSF1R in the human genome lead to the development of ALSP, which can be diagnosed by genetic screening of the blood. ALSP makes patients’ judgment, insight, memory, personality, executive function ability, social ability and motor ability have varying degrees of defects or changes, and eventually lead to patients’ spasticity and stiffness. Consistently, the authors found that a decrease in microglia in the white matter was associated with hypermyelination and eventual demyelination. These results demonstrate that microglia prevent myelin degeneration and that a lack of microglia causes demyelination in the central nervous system.

**Fig. 1:**
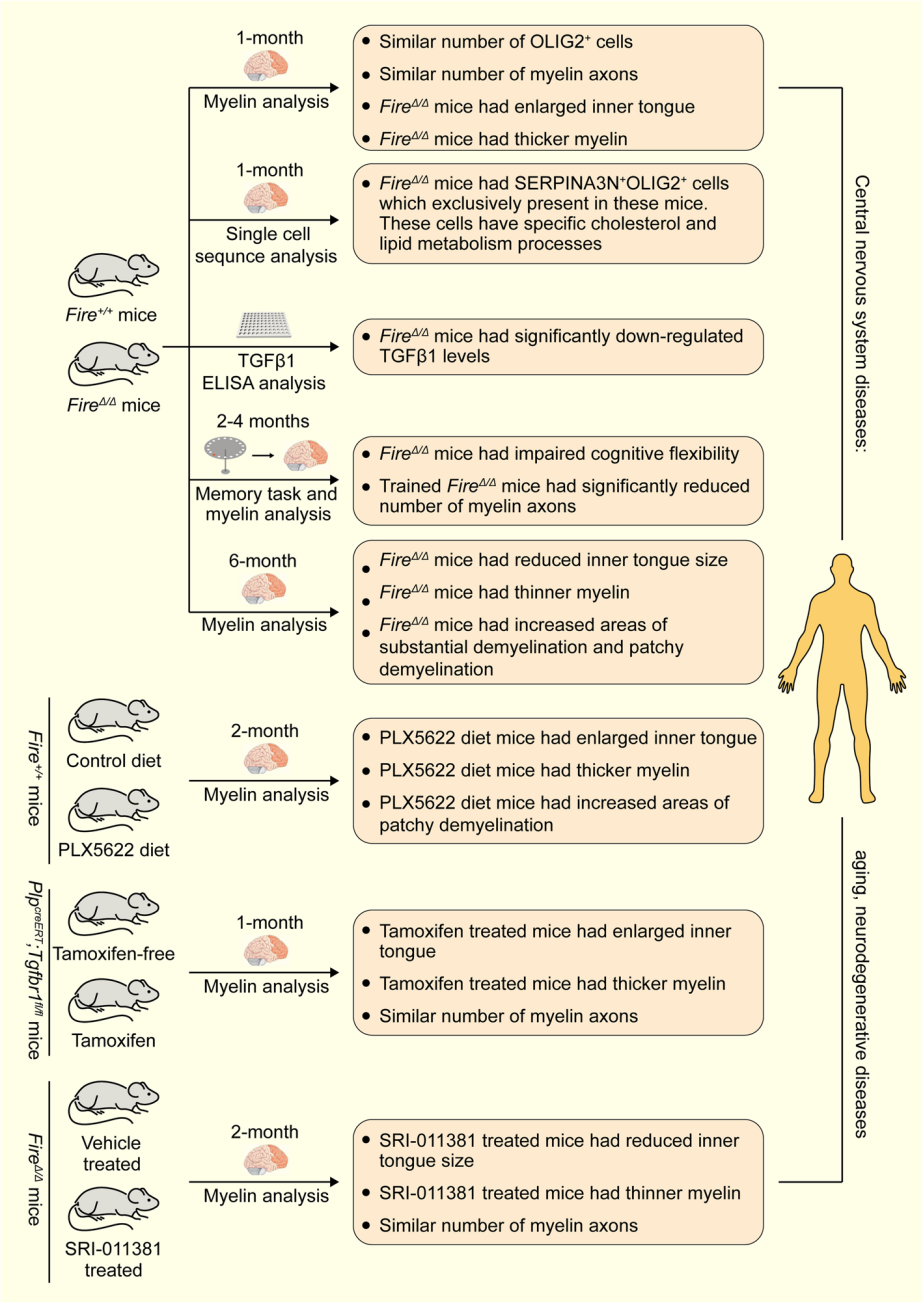
Microglia regulate myelin health in four mouse models. Diagram of four mouse models showing microglia regulating myelin health, including *Fire*^*+/+*^ and *Fire*^*Δ/Δ*^ mice model, control diet and PLX5622 diet *Fire*^*+/+*^ mice model, tamoxifen-free and tamoxifen *Plp*^*creERT*^;*Tgfbr1*^*fl/fl*^ mice model, and vehicle treated and SRI-011381 treated *Fire*^*Δ/Δ*^ mice model

To explore the cellular and molecular pathways by which microglia regulate myelin health, the authors conducted single-cell transcriptomic sequencing of 1-month mouse brain samples. SERPINA3N^+^OLIG2^+^ cells were almost exclusively present in the white matter of *Fire*^*Δ/Δ*^ mice and have specific lipid synthesis and metabolism processes. It has been reported that TGFβ1 is mainly expressed by microglia in mice and human brains, and it affects lipid metabolism. The authors found that TGFβ1 levels in *Fire*^*Δ/Δ*^ mice were significantly downregulated. Because loss of *Tgfb1* in the central nervous system can lead to changes in the number and homeostasis of microglia cells and infiltration of monocytes, the authors further chose to perform conditional knockout of *Tgfbr1* in mature oligodendrocytes (*Plp*^*creERT*^;*Tgfbr1*^*fl/f*l^). The authors found that *Tgfbr1* conditional knockout resulted in enlarged inner tongues on smaller diameter axons and thicker myelin on larger diameter axons. Finally, the authors used SRI-011381 hydrochloride, a small-molecule activator of the TGFβ signaling pathway, to investigate whether myelin abnormalities in *FireΔ/Δ* mice could be rescued by targeting the TGFβ1-TGFβR1 signaling pathway. The results showed that SRI-011381 significantly reduced the thickness of the inner tongue and myelin in *Fire*^*Δ/Δ*^ mice. These results suggest that the loss of microglial cells leads to the emergence of oligodendrocytes with abnormal lipid metabolism through the TGFβ1-TGFβR1 signaling pathway, which ultimately leading to damage to the health of the myelin sheath.

These findings highlight the ability of microglia as a potential therapeutic target in the regulation of cognitive aspects, such as aging and other neurodegenerative diseases. These results also suggest that CSF1R inhibitors which currently in clinical or preclinical use should be used with caution and monitored for potential off-target effects on myelin health. Moreover, the discovery of changes in cholesterol and lipid metabolism in oligodendrocytes may reveal the underlying pathological mechanism of cholesterol ester increase in human nervous system diseases.

It is important to consider the transcriptome heterogeneity of microglia, such as development, homeostasis, demyelination, and age, to determine whether specific microglial states are required to regulate myelin integrity. It is also necessary to examine the compensatory role of perivascular macrophages, astrocytes and other glial cell types in myelination when microglia cells are absent. In addition, oligodendrocyte-related signaling pathways are down-regulated after up-regulation in the presence of large amounts of β-amyloid [5]. Whether this change represents a change in myelin remains to be explored. In conclusion, the authors determine that microglia are required to maintain a healthy central nervous system and that they provide possible therapeutic targets for the treatment of aging and neurodegenerative diseases.

## Data Availability

Not applicable.
